# Molecular Mechanisms of Inherited Arrhythmias

**DOI:** 10.2174/138920208784340768

**Published:** 2008-05

**Authors:** Cordula M Wolf, Charles I Berul

**Affiliations:** Department of Cardiology, Children’s Hospital Boston, Department of Pediatrics, Harvard Medical School, Boston, MA, USA

## Abstract

Inherited arrhythmias and conduction system diseases are known causes of sudden cardiac death and are responsible for significant mortality and morbidity in patients with congenital heart disease and electrical disorders. Knowledge derived from human genetics and studies in animal models have led to the discovery of multiple molecular defects responsible for arrhythmogenesis. This review summarizes the molecular basis of inherited arrhythmias in structurally normal and altered hearts.

On the cellular and molecular levels, minor disturbances can provoke severe arrhythmias. Ion channels are responsible for the initiation and propagation of the action potential within the cardiomyocyte. Structural heart diseases, such as hypertrophic or dilated cardiomyopathies, increase the likelihood of cardiac electrical abnormalities. Ion channels can also be up- or down-regulated in congenital heart disease, altering action potential cellular properties and therefore triggering arrhythmias. Conduction velocities may be inhomogeneously altered if connexin function, density or distribution changes.

Another important group of electrophysiologic diseases is the heterogeneous category of inherited arrhythmias in the structurally normal heart, with a propensity to sudden cardiac death. There have been many recent relevant discoveries that help explain the molecular and functional mechanisms of long QT syndrome, Brugada syndrome, catecholaminergic polymorphic ventricular tachycardia, and other electrical myopathies. Identification of molecular pathways allows the identification of new therapeutic targets, for both disease palliation and cure. As more disease-causing mutations are identified and genotypic-phenotypic correlation is defined, families can be screened prior to symptom-onset and patients may potentially be treated in a genotype-specific manner, opening the doors of cardiac electrophysiology to the emerging field of pharmacogenomics.

## INTRODUCTION

Inherited arrhythmias can be life threatening, and are major cause of mortality and morbidity in developed nations. Identification of molecular pathways that increase susceptibility to arrhythmia is necessary to prevent disease occurrence, to improve current therapies and to target new drug development. In recent years, the discovery of pathogenic mutations in inherited arrhythmia syndromes has provided novel insights for the understanding and treatment of diseases predisposing to sudden cardiac death. In patients with the long QT syndromes (LQTS), genotype-phenotype relation studies [1] and genetic testing have influenced patient risk stratification [2] and refined treatment strategies [3].

Arrhythmia mechanisms include abnormal automaticity, triggered activity, and re-entrant excitation. Each of these mechanisms can occur in any type of myocardial disease or in inherited cardiac arrhythmias. The current article focuses on molecular mechanisms of arrhythmias in the structurally abnormal and normal heart. Hypertrophic and dilated cardiomyopathies, as well as arrhythmogenic right ventricular dysplasia/cardiomyopathy are common substrates of inherited arrhythmias in the structurally abnormal heart. Genetic diseases causing arrhythmias in the structural normal heart, also called electrical myopathies, include the long QT syndromes, Brugada syndrome, catecholaminergic polymorphic ventricular tachycardia (CPVT), and non-defined familiar idiopathic ventricular fibrillation. Most, but not all of these disorders are caused by mutations in genes encoding cardiac ion-channel proteins. Among family members carrying an identical mutation in a single gene, remarkable phenotypic variability and expressivity may be observed, suggesting both environmental [4] and genetic modifiers [5].

## BACKGROUND

Fig. (**1A**) summarizes the action potential in a non-pacemaker cardiomyocyte and the contribution of distinct ion channels to the action potential shape. Voltage-gated Na^+^ channels are essential for the amplitude and upstroke velocity of the cardiac action potential, which are important determinants for impulse propagation and conduction velocity throughout the fast conducting conduction system and the working myocardium [6]. Potassium (K^+^) channels control resting potentials, action potential waveforms, automaticity, and refractory periods [7]. Calcium (Ca^2+^) channels contribute to the plateau phase of the action potential. In the past decade, numerous genes encoding various ion channels have been cloned and their structural biophysical properties, subunit stoichiometry, channel assembly and modulation by intracellular second messengers and ligands have been characterized [8] (see Fig. **2**). 

There are three different mechanisms that lead to tachyarrhythmias: Increased automaticity of myocytes, triggered activity arising from early and delayed afterdepolarizations, and re-entry (see Fig. **3**). Increased automaticity and triggered activity are the typical mechanisms of non-re-entrant tachycardias [9].

Increased automaticity of a myocyte denotes that the myocyte is able to depolarize itself, without an external stimulus. Normally, there is a stable resting membrane potential in a cardiomyocyte. Only pacemaker cardiomyocytes depolarize in the absence of extrinsic stimulation. Those automatic cells spontaneously depolarize during phase 4 of the cardiac action potential, reach threshold, and initiate an action potential.

In contrary to increased automaticity where focal activity starts spontaneously, “triggered” rhythms typically require a preceding action potential. Thus, triggered activity means that arrhythmias originate from afterdepolarizations. An afterdepolarization is a premature depolarization of the membrane potential before the ongoing action potential has reached the next phase 0. Afterdepolarizations arise as a consequence of intracellular calcium oscillations.

**Fig. (1). Action potential in non-pacemaker cardiomyocytes.**
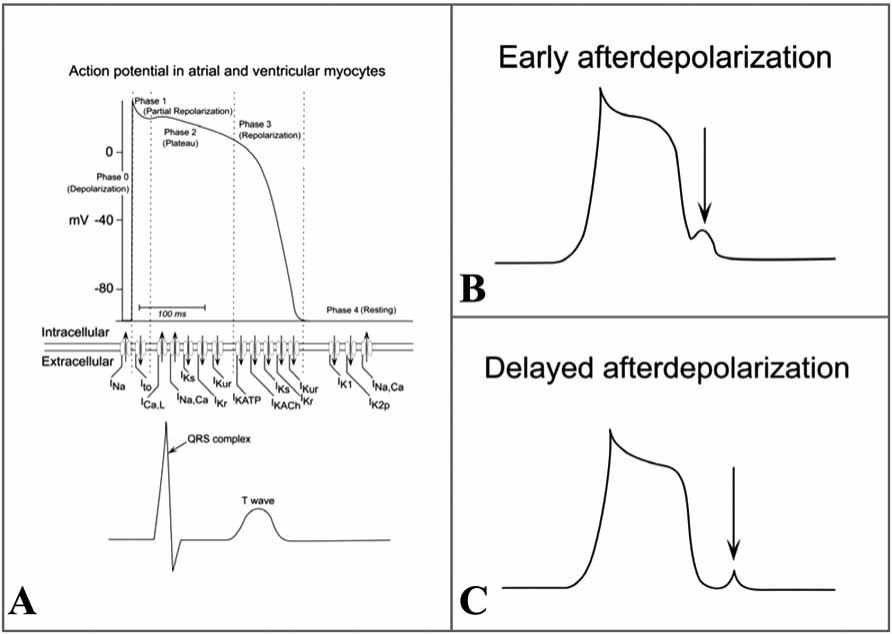
**A**). The action potential of atrial and ventricular myocytes consists of 4 phases (upper part), to which specific ion flows contribute (middle). The long plateau phase (3) and a stable resting phase (4) are characteristic for myocyte action potential. The corresponding electrocardiographic ventricular activity is shown below. Early afterdepolarizations occur during phase 2 / 3 of the action potential, before the ongoing action potential has reached phase 0 (**B**). Delayed afterdepolarizations occur during phase 4 of the action potential, before the action potential has reached phase 0 (**C**).

Early afterdepolarizations occur during phase 2 or 3 (repolarization phase), whereas delayed afterdepolarizations occur during phase 4 (resting phase) (see Fig. **1**). If an afterdepolarization is large enough, it can engage rapid sodium influx and thus initiate a premature action potential, which is clinically observed as a premature contraction.

Another common arrhythmia mechanism is re-entrant excitation. Re-entry contributes to the development of ventricular tachycardia, a rapid, organized process in which the excitation travels a relatively well-defined circuit. Micro-re-entrant circuits involve small regions of heart tissue such as seen in LQT and Brugada syndrome. Here, intramural reentry is caused by a transmural dispersion of refractoriness. Macro-re-entry involves large regions of heart tissue and is most commonly seen in hypertrophied or stretched myocardium as seen in cardiomyopathies or in congenital heart disease (see Fig. **3**). 

Re-entry can degenerate to atrial or ventricular fibrillation, a chaotic electrical activity resulting from random propagation of multiple independent wavelets through the muscle. Fibrillation results in unorganized impulse propagation and absent contraction.

## ARRHYTHMIA MECHANISMS IN THE STRUCTURALLY ALTERED HEART

### Hypertrophic and Dilated Cardiomyopathies

Both hypertrophic cardiomyopathies (HCM) and dilated cardiomyopathies (DCM) are inherited disorders with a high incidence of lethal arrhythmias occurring at a young age. In both HCM and DCM, arrhythmias may occur unrelated to ischemia or heart failure.

HCM is a disease of the sarcomere and is the most common inherited cardiovascular disorder with a prevalence of 0.2-0.5% in the general population [10-12]. Sudden death, at least partially due to malignant arrhythmias, accounts for at least 50% of deaths among patients with HCM [13].

The actual mechanisms linking contractile dysfunction and arrhythmias remain poorly understood [14]. Multifactorial biological substrates including genetic, functional, environmental and hemodynamic features contribute to a high incidence of lethal ventricular tachycardias in cardiomyopathies. Histopathologic changes, such as myocardial fibrosis or myocyte disorganization and hypertrophy, might attribute to re-entry and thus engender arrhythmia susceptibility [15, 16]. However, only ventricular hypertrophy and not histopathologic changes, correlated with increased arrhythmia susceptibility in a HCM mouse model [17]. Increased automaticity by upregulation of pacemaker ion channel genes, which has been shown to occur in cardiac hypertrophy [18], could be another arrhythmia mechanism in HCM. Furthermore, sarcomere gene mutations alter cytosolic cardiomyocyte Ca^2+^ homeostasis [19] which promotes afterdepolarizations triggering arrhythmias [20] (see Fig. **3**).

DCM is characterized by an increase in size of the ventricular chambers and frequently is associated with arrhythmias. Numerous mutations affecting the sarcomere and the cytoskeleton have been identified in DCM [21, 22]. The etiology of arrhythmias occurring in DCM is poorly understood, but it is known that stretch of the myocardium per se influences the shape and amplitude of the intracellular Ca^2+^ transient, therefore favoring afterdepolarizations [23].

DCM caused by mutations in the *LMNA* gene encoding for the inner nuclear membrane protein lamin A/C is associated with a high risk for arrhythmias and sudden death [24-26] and sometimes involves progressive atrioventricular nodal disease [26-28]. Both arrhythmias and conduction system disease can precede cardiac dilatation and presumably are a direct consequence of intrinsic changes of the myocyte secondary to lamin A/C deficiency [29]. 

### Arrhythmogenic Right Ventricular Dysplasia/Cardiomyopathy (ARVD/C)

ARVD/C refers to a genetically heterogeneous group of cardiomyopathies characterized by progressive degeneration and fibrofatty infiltration of the right ventricular myocardium [30]. Patients are prone to ventricular tachycardia, right heart failure, and sudden death [31, 32]. Mutations in genes encoding for the desmosomal proteins plakoglobin [33], desmoplakin [34, 35], and plakophilin-2 [36] have been shown to cause ARVD/C. The molecular mechanisms of arrhythmias in ARVD/C are poorly understood. ARVD2, caused by mutations in the ryanodine receptor (RyR2), is clinically different from other forms of ARVD/C in that ventricular arrhythmias are stereotypically effort-induced [30]. In ARVD2 the leaky ryanodine receptor results in cytosolic Ca^2+^ overload and, consequently, in delayed afterdepolarizations triggering arrhythmias, and may overlap with CPVT.

### Arrhythmia mechanisms in the structurally normal heart

The occurrence of arrhythmias in the absence of any structural heart disease is classified as a “primary electrical disease”. Ion channelopathies are examples of arrhythmogenic mechanisms generated primarily at the cellular level. It is still unclear how arrhythmias are triggered at a multicellular level, but the presumption is that delayed repolarization increases the spatial voltage gradients sufficiently to trigger repetitive excitation of the ventricle. A defect in an ion channel alone is not sufficient to induce arrhythmias. Extensive phenotypic variability can be seen among family members carrying mutations in these channel genes. While some family members harboring the mutations suffer from sudden cardiac death, other members carrying the same primary genetic mutations may never develop any arrhythmias. The underlying cause for the onset of lethal arrhythmias must be therefore multifactorial.

### Long QT Syndromes

The long QT syndrome is characterized by the electrocardiographic finding of QT interval prolongation and T wave abnormalities in conjunction with a clinical presentation and/or a family history of syncope, ventricular arrhythmias, and unexpected sudden death. This genetic disorder of cardiac electrical repolarization is caused by mutations of genes encoding for cardiac potassium and sodium channels, usually inherited in an autosomal-dominant fashion. The altered ion channel function produces prolongation of the action potential and a propensity to torsades de pointes ventricular tachycardia. “Torsades de Pointes” means twisting around the point, an allusion to the alternating axis of the QRS complex around the isoelectric line of the ECG during this arrhythmia.

**Fig. (2). Cellular substrates for action potential initiation and propagation.**
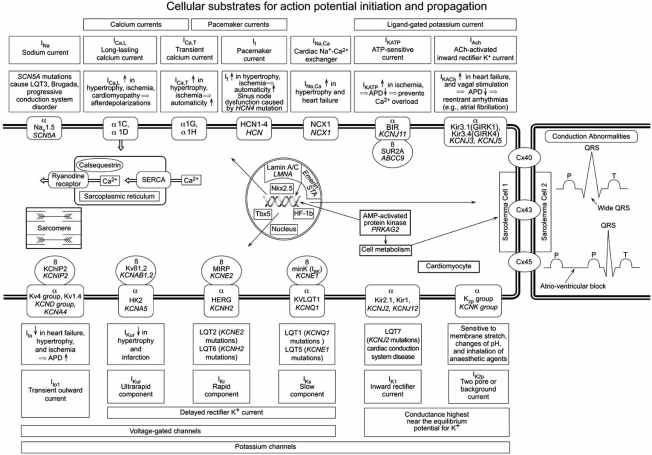
Displayed are transverse tubules of two neighboring cardiomyocytes with the ion channels localized on the sarcolemma of cell 1 and the connecting gap junction connexins to the sarcolemma of cell 2. Shown are the pore regions of the ion channels (α-subunits) through which ions flow across the plasma membrane, and the cytoplasmic β-subunits. Within each subunit, the encoding gene is displayed in italics, the protein in normal font. Differences in disease status are indicated in a box next to the currents. Intranuclear proteins and genes might also interact with ion channels and/or gap junction proteins. Disturbed calcium handling within the sarcomere or sarcoplasmic reticulum underlies distinct arrhythmia causing diseases.

**Fig. (3). Cellular mechanisms of arrhythmias.**
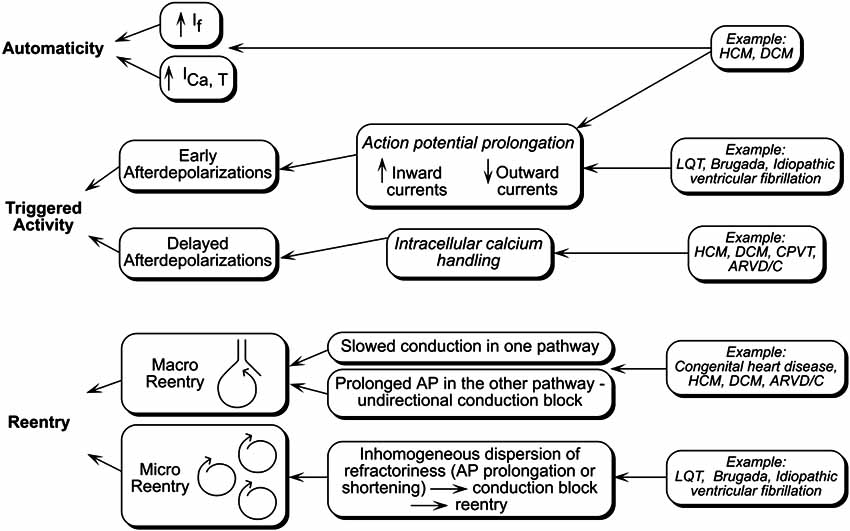
On the cellular level, three distinct entities causing tachyarrhythmias can be distinguished: automaticity, triggered activity and reentry. One or a combination of these mechanisms occurs in inherited arrhythmias. AP action potential, HCM hypertrophic cardiomyopathy, DCM dilated cardiomyopathy, ARVD/C arrhythmogenic right ventricular dysplasia/cardiomyopathy.

To date, more than ten different types of LQTS have been characterized according to their underlying monogenic defect (see Table **1** and Fig. **2**).

LQT1 (42%) and LQT2 (45%) account for about 87% of identified mutations, and LQT3 (8%), LQT5 (3%), and LQT6 (2%) for the other 13% [37]. Missense mutations in the transmembrane and pore domains are the most common [37]. Mutant subunits lead to reduction of I_Ks_ or I_Kr_ by a loss-of-function mechanism, often with dominant-negative effect [38-41]. 

LQT syndrome-associated mutations in *SCN5A* cause a gain-of-function leading to prolonged opening of the sodium channel [42, 43]. 

In the heart, reduced I_Ks_ or I_Kr_ or increased I_Na_ leads to prolongation of the cardiac action potential, lengthening of the QT interval, and increased risk of ventricular arrhythmia (see Fig. **2**).

Genes such as *KVLQT1, HERG, SCN5A, KCNE1, *and* KCNE2 *have been implicated in the autosomal-dominant Romano-Ward syndrome, which is associated with long QT syndrome in the absence of other phenotypic abnormalities [44]. Mutations in *KVLQT1* and *KCNE1* also cause the less-common autosomal recessive Jervell and Lange-Nielsen syndrome [45, 46], characterized by congenital bilateral deafness associated with marked QT prolongation on the electrocardiogram, syncopal attacks due to ventricular arrhythmias and a high risk for sudden death. 

Recent finding of a mutation in the *ANK2* gene encoding for ankyrin-B [47] at the LQT4 locus reveals that the phenotype of LQTS can also be caused by abnormal proteins other that cardiac ion channels. *ANK2 *mutations modify ankyrin-B binding proteins and alter the sodium pump [48], inositol-1,4,5-triphosphate receptors, and the sodium/calcium exchanger function, thereby increasing arrhythmia susceptibility [47]. 

LQT7, or Andersen syndrome (And1), describes a clinical disorder consisting of potassium-sensitive periodic paralysis, ventricular arrhythmias, and dysmorphic features [49], caused by a missense mutation in the *KCNJ2* gene [50].

Timothy's syndrome presents LQT8 and is due to mutations in the calcium channel Cav1.2 encoded by the gene *CACNA1c*. Since the Calcium channel Cav1.2 is abundant in many tissues, patients with Timothy's syndrome have many clinical manifestations including congenital heart disease, autism, syndactyly and immune deficiency [51].

Most recently mutations in the gene *CAV3 *encoding for Caveolin-3, a structural protein, have been described to cause LQT9 [52]. Caveolin-3 is the major scaffolding protein forming specific membrane domains in the heart called caveolae and. Caveolin-3 co-localizes and interacts with the *SCN5A* sodium channel, thereby altering ion channel function and presumably increasing arrhythmia vulnerability [52].

Last, the Na^+^ channel ß4 subunit encoded by *SCN4B* has been identified as a novel and rare LQT-susceptibility gene (LQT10). A missense mutation in *SCN4B* was found to cause a secondary gain of function on the Na^+^ channel mimicking that of classic LQT3-associated mutations in *SCN5A *[53].

Understanding the molecular differences between the different LQT types is important for risk stratification and has clinical implications for the management of LQT patients. Genetic screening using mutational analysis can improve presymptomatic diagnosis and has recently become commercially available.

A large phenotype-genotype correlation study showed that LQT1 patients are more prone to arrhythmias during exercise and swimming, whereas LQT2 and LQT3 patients experience more lethal and non-lethal events during rest and sleep [1]. The response to medical therapy differs between underlying LQT mutation: LQT1 patients seem to respond better to beta-blocker therapy than LQT2 and LQT3 patients [1, 3]. 

However, clinical heterogeneity among patients with long-QT syndrome sharing the same disease-causing mutation challenges their medical management. Some recognized modifiers of the clinical phenotype include gender, age, drugs, metabolic derangements or hypothermia. Additionally, recent studies provided more evidence for genetic modulation altering arrhythmia susceptibility in primary arrhythmia syndromes, such as the presence of compound mutations [54] or the coexistence of modifier alleles or single nucleotide polymorphisms [55].

Modifier genes also play a significant role in drug-induced LQT syndrome. There are differences in responses of patients to medication and knowing the gene variants that cause differences among patients allow ‘personalized’ drug therapy [56]. This is of specific interest regarding drugs that are known I_Kr_ blockers, such as certain antibiotics, antihistamines, antipsychotics and antiarrhythmic agents [57].

### Brugada Syndrome

Brugada and Brugada described an autosomal-dominant life-threatening disease occurring in the structurally normal heart and characterized by ST segment elevation in the right precordial leads (V1 to V3), right bundle branch block, and susceptibility to ventricular tachyarrhythmias [58]. The cellular basis for the Brugada syndrome is thought to be due to an outward shift in the ionic current active during phase 1 of the right ventricular epicardial action potential [59] in which I_to_ is prominent, exaggerating shortening of action potential duration in epicardium versus endocardium [60].

Mutations in the *SCN5A* gene account for less than 25% of cases [61]. In contrast to LQT3, these mutations result in a loss-of-function of I_Na_ [62] (see Fig. **2**).

Recently, a novel mutation in the glycerol-3-phosphate dehydrogenase 1–like gene (*GPD1-L*) was identified to cause Brugada syndrome. By disrupting trafficking of *SCN5A*, *GPD1-L* mutations decrease *SCN5A* surface membrane expression and reduces I_Na_ [63].

### Catecholaminergic Polymorphic Ventricular Tachycardia

Arrhythmias in catecholaminergic polymorphic ventricular tachycardia (CPVT) are typically triggered by catecholamine surges as occurs with exercise and emotion [64]. An alternating QRS axis on a beat-to-beat basis, the so-called bidirectional VT, is often the distinguishing pathognomonic presentation of CPVT-related arrhythmias [65]. Supraventricular arrhythmias are also frequently observed during exercise among CPVT patients.

Mutations in the ryanodine receptor gene (*RyR2*) [65, 66] and the calsequestrin gene *CASQ2 *[67], both involved in intracellular calcium homeostasis and excitation-contraction coupling, are two of the genetic causes of CPVT (see Fig. **2**). 

The underlying pathogenic mechanism of arrhythmias in both ryanodine [68] as well as calsequestrin gene mutations [69] is a leakiness of the ryanodine receptor causing intracellular Ca^2+^ disturbances. Catecholamine surges caused by stress or exercise furthermore increase intracellular calcium overload and thus trigger afterdepolarizations, resulting in polymorphic ventricular tachycardia.

### Idiopathic Ventricular Fibrillation

Variable penetrance and distinct modifiers in allelic disorders make disease definitions challenging. If sudden unexpected cardiac death occurs and no electrophysiological abnormality is recognized, this patient group is categorized as having idiopathic ventricular fibrillation. The terminology acknowledges the current inability to identify a specific cause [70]. Patients who survive an episode of ventricular fibrillation are at a high risk of recurrence for life-threatening events [71]. 

In some patients with idiopathic ventricular fibrillation, mutations in the sodium channel encoding gene *SCN5A* have been revealed [72, 73], but a subset of these also present a Brugada or LQT like phenotype [74].

Sudden unexplained death syndrome (“SUDS”, or “Lai-Tai” in Thai) is more prevalent in the Asian as compared to the western population. The cause of this disorder is widely unknown. Most patients suffering from SUDS show Brugada-like ECG pattern, but mutations in the gene were found in a minority of them [75]. Therefore, *SCN5A* mutations different from previously reported ones or other candidate genes might be responsible for SUDS and remain to be investigated.

Recently, genome-wide association studies have found a common variant in the nitric oxide synthase 1 adaptor protein (*NOS1AP*) to be associated with QT-interval variation [76] but increased risk for ventricular fibrillation and sudden death could not yet be established [77].

## FUTURE

Understanding of the molecular and ionic mechanisms underlying cardiac electrophysiology is essential for the appreciation of the pathogenesis of cardiac arrhythmias in structurally normal and altered hearts. These fundamental principles and concepts allow incorporation of genetic factors in addition to clinical parameters to ultimately improve personalized risk assessment and determine optimal therapy [14]. 

One area in which technologies are rapidly evolving is that of polymorphism identification. These data will be used to identify single nucleotide polymorphisms, or groups of polymorphisms, that predict particular diseases or individualized responses to drug therapies. 

Another new therapeutic field is the gene therapy approach. Gene delivery and cell-based therapies are currently being explored for treating the substrate for re-entry after myocardial infarction or for replacing electronic pacemakers with biological ones [78, 79]. In the experimental setting, over-expression of β2-adrenergic receptors [80], use of a dominant-negative construct to suppress inward rectifier current when expressed together with the wild-type Kir2.1 [81], and implantation of vectors carrying the pacemaker gene *HCN2* into atrium [82] or bundle branches [83] have been studied. A common problem inherent in these approaches is the use of viruses to deliver the necessary genes. Although the vectors are replication-deficient adenoviruses that have little infectious potential, there is concern related to the possibility of only a transient improvement in pacemaker function as well as potential inflammatory responses [84].

The identification of molecular pathways allows development of new therapeutic targets, for both disease palliation and cure. Increasing knowledge about disease causing mutations and genotypic-phenotypic correlation opens the doors of cardiac electrophysiology to the emerging field of pharmacogenomics.

## Figures and Tables

**Table 1. Long QT Syndromes:** More than ten distinct types of congenital long QT (LQT) syndromes due to different gene mutations have been defined to date. Most LQT syndromes (LQT1, 2, 5, 6 and 7) are caused by loss-of-function mutations in potassium channels. LQT3 and LQT10 involve mutations in the sodium channel. LQT8 is caused by mutations in the L-type calcium channel. LQT4 and LQT9 are caused by structural proteins.

	Gene Mutations	Affected Protein/Subunit	Ion Channel
LQT1	*KCNQ1*	KVLQT1 Kv7.1α	I**_Ks_**
LQT2	*KCNH2*	HERG Kv11.1α	I**_Kr_**
LQT3	*SCN5A*	Na_v_1.5α	I**_Na_**
LQT4	*Ank2*	Ankyrin-B	-
LQT5	*KCNE1*	MinKβ	I**_Ks_**
LQT6	*KCNE2*	MiRP1β	I**_Kr_**
LQT7 (Andersen syndrome)	*KCNJ2*	Kir2.1α	I**_K1_**
LQT8 (Timothy syndrome)	*CACNA1c*	Cav1.2α1c	I**_Ca,L_**
LQT9	*CAV3*	Caveolin-3	-
LQT10	*SCN4B*	Na_v_1.5β4	I**_Na_**
